# Haplotypes of the Mutated SIRT2 Promoter Contributing to Transcription Factor Binding and Type 2 Diabetes Susceptibility

**DOI:** 10.3390/genes11050569

**Published:** 2020-05-19

**Authors:** Xiao Zheng, Jiajun Li, Jie Sheng, Yang Dai, Yue Wang, Jinbiao Liu, Yao Xu

**Affiliations:** 1 Institute of Biology and Medicine, College of Life Sciences and Health, Wuhan University of Science and Technology, Wuhan 430081, China; Zx13277957103@163.com (X.Z.); ljj13122872601@163.com (J.L.); shengj2019@163.com (J.S.); daiyang2015@163.com (Y.D.); wustwangyue@163.com (Y.W.); 2 Institute of Medical Microbiology, Jinan University, Guangzhou 510632, China

**Keywords:** type 2 diabetes, *SIRT2* gene, haplotype combination, association, transcriptional activity

## Abstract

Genetic variability is an important causative factor for susceptibility and pathogenesis of type 2 diabetes (T2D). Histone deacetylase, sirtuin 2 (SIRT2), plays regulatory roles in glucose metabolism and insulin sensitivity. However, whether the *SIRT2* variants or haplotypes contribute to T2D risk remain to be elucidated. In this study, we first detected three novel polymorphisms (P-MU1, P-MU2, and P-MU3) in the promoter of *SIRT2* in the Chinese population. All pairwise sets of the three loci were strongly in linkage disequilibrium. Next, we constructed the haplotype block structure, and found H1-GGC and H2-CCA accounted for the most (total 91.8%) in T2D. The haplotype combination H1-H1-GGGGCC displayed a high risk for T2D (OR = 2.03, 95% CI = 1.12–3.72). By association analysis, we found the individuals carrying H1-H1-GGGGCC had significantly higher fasting plasma glucose and glycated hemoglobin. The haplotype H1-GGC presented a 6.74-fold higher promoter activity than H2-CCA, which was consistent with the correlation results. Furthermore, we clarified the mechanism whereby the C allele of both the P-MU1 and P-MU2 loci disrupted the signal transducer and activator of transcription 1 (STAT1) binding sites, leading to the attenuation of the *SIRT2* transcription. Together, these data suggest that the linked haplotype GGC could be considered as a promising marker for T2D diagnosis and therapy assessment.

## 1. Introduction

Epidemiological studies have revealed that the rising incidence of endocrine and metabolic disorders, including diabetes, has significantly increased worldwide in recent years [1]. Type 2 diabetes mellitus (T2D) is an epidemic, complex, and multifactorial diabetes in human beings, and an abnormal glucose concentration and insulin resistance are the major predictors of the pathogenesis and development for T2D and its complications [2]. Insulin, secreted from pancreatic β cells, plays important roles in the regulation of uricemia, lipidemia, and glycemia [3]. Accordingly, insulin resistance can lead to elevated insulin and hyperinsulinemia in a compensatory manner. Whole genome sequencing and genome-wide association studies have identified thousands of genetic variants from T2D patients, some of which are associated with insulin sensitivity and secretion [4,5], suggesting that identification of genetic heterogeneity is a crucial step in the diagnosis, prevention, and therapy of T2D etiologies.

Mammalian sirtuins (SIRTs) belong to a conserved family of nicotinamide adenine dinucleotide (NAD^+^)-dependent histone deacetylases, in which seven homolog family members have been identified (SIRT1-7) [6]. Continuously increasing evidence indicate that SIRTs display a diversity of metabolic tissues and participate in a myriad of biological processes, such as energy homeostasis, oxidative stress pathways, and metabolism [7,8]. SIRT2, localized in both the nucleus and cytoplasm, belongs to a separate class of deacetylases (class III) that modify lysine on histone as well as many functional proteins. Recently, SIRT2 was reported to be implicated in sustaining metabolic homeostasis, including adipocyte differentiation, gluconeogenesis, and insulin sensitivity [9]. In T2D disorders, AKT kinase was the main factor involved in insulin resistance. As a novel AKT interactor, SIRT2 overexpression significantly enhanced the AKT activation and its downstream targets [10]. In addition, SIRT2 expression was positively correlated with body mass index and high density lipoprotein cholesterol in children with obesity and insulin resistance [11]. Defining the role of SIRT2 in the cytosolic acetylation state, SIRT2 knockout mice demonstrated an increase in body weight due to impaired insulin action, and this effect was exacerbated in high-fat-fed mice [12]. Given the fact that SIRT2 plays a critical role in metabolic activities, *SIRT2* could be suggested as a promising causative gene for the pathogenesis and development of T2D.

Numerous studies revealed that genetic mutations of functional genes, especially those located in the regulatory and coding regions, may affect the gene structure and expression level, finally contributing to the diabetic pathogenesis and susceptibility [13,14]. Haplotype blocks (multiple SNPs) are emerging to be more effective and precise in their application of disease diagnosis and therapy due to linkage disequilibrium of the SNPs in chromosomes compared to a single SNP [15]. In this study, we aimed to detect the novel SNP mutation in the *SIRT2* gene promoter region in the Chinese population, and to investigate the effects of the SNP haplotype combinations on the promoter activity and clinical characteristics of T2D patients, and finally to elucidate the molecular mechanism of the genetic variation, which may provide convincing evidence for the extensive usage of haplotypes.

## 2. Materials and Methods

### 2.1. Study Participants

A total of 209 T2D patients from the affiliated Tianyou Hospital of Wuhan University of Science and Technology were enrolled in the present study from May 2015 to June 2016. The clinical diabetic conditions were diagnosed according to the criteria of the World Health Organization (WHO) National Diabetic Group criteria of 2006, as follows: the plasma glucose concentration was ≥11.1 mmol/L after 2 h for an oral glucose tolerance test (OGTT), and the fasting plasma glucose was ≥7.0 mmol/L [16]. A total of 223 nondiabetic subjects were enrolled from the population undergoing routine health checkups at the Hospital of Wuhan University from May 2015 to March 2016. All participants were long-term residents in the Hubei province, and they have no second-degree or closer relationships. All subjects gave their informed consent for inclusion before they participated in the study. This study was conducted in accordance with the Declaration of Helsinki, and the protocol was approved by the Ethics Committee of the affiliated Tianyou Hospital of Wuhan University of Science and Technology (no. HCTY-2017-11-106). Genomic DNA was extracted from the peripheral leukocytes of all subjects by phenol-chloroform extraction.

### 2.2. Clinical Parameters and Serum Biomarkers

Anthropometric characteristics, including height (m) and weight (kg), were measured using standard methods [17]. Body Mass Index (BMI) was directly calculated as the ratio of the weight divided by the square of the height. All biochemical parameters were measured according to the standard protocols, and the biological analysis was carried out in a Hitachi 912 Autoanalyzer (Roche, Mannheim, Germany) according to the manufacturer’s instructions. For instance, fasting plasma glucose was analyzed by the glucose oxidase–peroxidase method. Glycerol phosphate oxidase peroxidase–amidopyrine and cholesterol oxidase peroxidase–amidopyrine were used to detect serum triglycerides and total cholesterol, respectively. High density lipoprotein (HDL) cholesterol and low density lipoprotein (LDL) cholesterol were estimated by Friedewald formula [18]. Levels of glycated hemoglobin (HbA1c) was measured by HPLC (Bio Rad, Hercules, CA). In addition, the OGTT assay was conducted in T2D populations for evaluating the glucose metabolism function. During the three days preceding the OGTT, all patients undergo normal diets and avoid high sugar and high carbohydrate intake, and then each participant sustained a 12-h overnight fasting and ingested 75 g glucose. Whole blood samples were adopted before (0 min) and after ingesting the glucose for 60, 120, and 180 min, respectively. Plasma glucose and insulin were measured using standard methods, as described earlier [19]. All clinical measurements were collected and summarized in Table S1.

### 2.3. SNP Genotyping Assay

The extracted genomic DNA from all study cohorts was quantified using NanoDrop One (ThermoFisher, Waltham, MA), and the concentration was diluted to 50 ng/μL. A fragment of 1675 bp (from −1782 to −108) within the human *SIRT2* gene promoter region was amplified and bi-directionally sequenced using an ABI PRISM BigDye Terminator v3.1 Cycle Sequencing kit and an Applied Biosystems 3730XL genetic analyzer (Applied Biosystems, Foster City, CA, USA). The sequenced PCR products were aligned with the *SIRT2* promoter sequences (NCBI, NC_000019.10) using a BLSTN assay. The identified SNP loci were genotyped by the PCR-restriction fragment length polymorphism (PCR-RFLP) method in this study. In detail, the *Msp* I-RFLP, *Alu* I-RFLP, and *Hin*f I-RFLP were established to genotype the P-MU1, P-MU2, and P-MU3 loci of the *SIRT2* gene, respectively. The primers that were used for detecting and base remodeling are listed in Table S2. More importantly, the accuracy of the RFLP genotyping assay was evaluated due to the concordance between duplicate samples (up to 100% for each SNP).

### 2.4. Linkage Disequilibrium and Haplotype Analysis

The SNPs loci of the *SIRT2* gene were screened by a minor allele frequency cutoff of 5% and analyzed using the correlation coefficient (r^2^) cutoff of 0.8 for linkage disequilibrium and 0.99 for complete linkage disequilibrium. Haploview software was used to calculate r^2^ and D’ as the measurements of linkage disequilibrium extent between pairwise SNP combinations. Haplotype combination blocks of different genotypes were determined using the SHEsis software (http://analysis.bio-x.cn/myAnalysis.php). Haplotype frequency as a measurement of genetic distribution was directly calculated in T2D and healthy control populations.

### 2.5. Promoter Activity Analysis with a Dual-Luciferase Reporter Assay

A series of promoter regions containing different *SIRT2* haplotypes were amplified from the genomic sequence of the corresponding study cohort and cloned into the luciferase reporter plasmid pGL3-Basic (Promega, Madison, WI, USA) by the *Nhe* I and *Hin*d III double digestion assay. The constructed plasmids were transfected into 293T cells to test the transcriptional activity. In detail, the 293T cells were cultured in a 48-well plate and transfected with 400 ng DNA containing the haplotype constructs and the internal control, pRL-TK (Promega, Madison, WI, USA), using the Lipfectamine^TM^ 3000 reagent (Invitrogen, Carlsbad, CA, USA). After transfection for 48 h, cells were collected, and the luciferase activity was detected using the Dual-Luciferase Reporter Assay System (Promega) following the manufacturer’s protocol. Promoter activity was determined as the values of firefly luciferase divided by *Renilla* luciferase. In addition, the promoter activity also was detected when the 293T cells were co-transfected with both a haplotype plasmid and si-STAT1 or the si-Control.

### 2.6. Chromatin Immunoprecipitation (ChIP) Assay

The ChIP assay was performed with the SimpleChIP Enzymatic Chromatin IP Kit from Cell Signaling Technology (Danvers, MA, USA). The 293T cells were transfected with pGL3-Basic plasmids containing different haplotypes. After 24 h, the cells were crosslinked by 1% formaldehyde for 10 min; DNA was then subjected to immunoprecipitation using antibodies against SIRT2 or non-specific IgG (Abcam). Finally, purified DNA was amplified by PCR with the primers: 5′-TGA GAATCATAGTTCAAGAA-3′ (P-MU1-forward) and 5′-TACTCCTAAATCTGACT TCC-3′ (P-MU1-reverse); 5′-GGAAGTCAGATTTAGGAGTA-3′ (P-MU2-forward) and 5′-TCTTCGGCTACGTCACTGAG-3′ (P-MU2-reverse). PCR products were then detected by agarose gel electrophoresis.

### 2.7. Statistical Analysis

The frequencies of genotypes, alleles, and haplotypes were directly calculated in T2D and healthy controls. Heterozygosity (He) and Polymorphism Information Content (PIC) were analyzed using the HET program. The genotypic frequencies of each SNP were measured for deviations from Hardy–Weinberg equilibrium within the population based on a χ^2^ test. The deviations of genotype distributions between the case and control were analyzed using the Crosstab of SPSS software (version 20.0, Illinois, USA). Association analyses were carried out for all clinical traits with SNP genotypes or haplotypes based on a general linear regression model, where the variables were adjusted for possible confounding factors, such as age, sex, and BMI, for normalization. A *P*-value less than 0.05 was considered a statistically significant difference.

## 3. Results

### 3.1. Discovery and Genotyping of the SIRT2 SNPs

**Figure 1:**
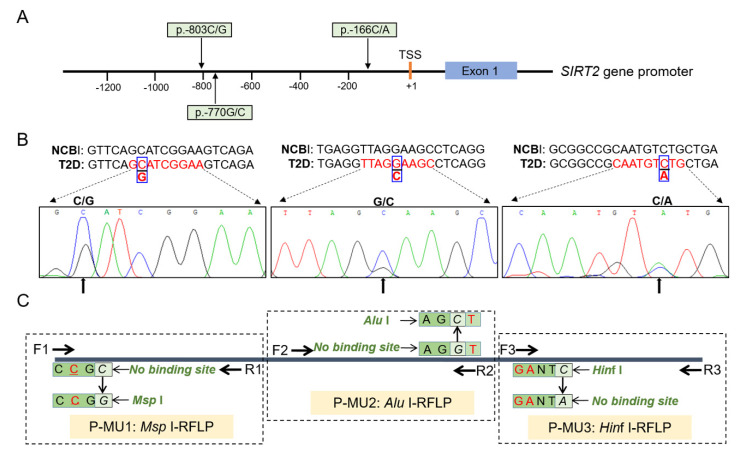
Discovery and genotyping of three SNPs in the *SIRT2* promoter region. (**A**) Location of the three novel SNPs found in our study. (**B**) Sequencing maps of the three SNPs. (**C**) Schematic diagram of genotyping the P-MU1, P-MU2, and P-MU3 loci by *Msp* I-RFLP, *Alu* I-RFLP, and *Hin*f I-RFLP, respectively.

**Figure 2:**
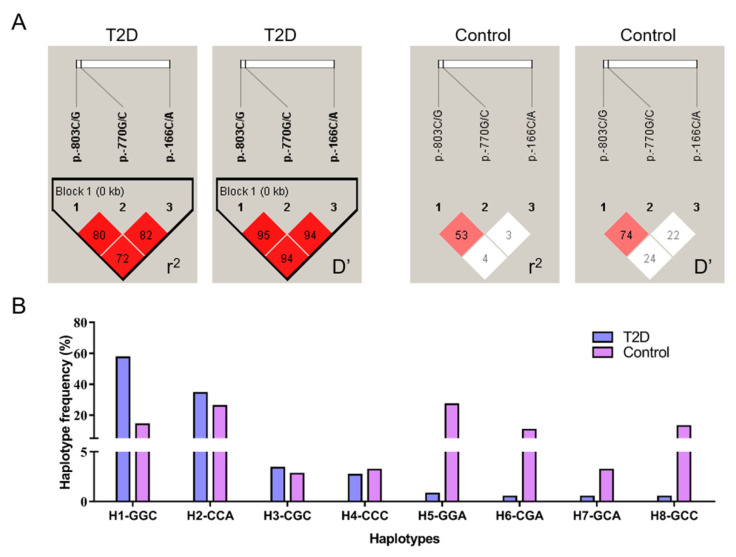
Linkage disequilibrium analysis and haplotype block construction of the *SIRT2* variants. (**A**) Linkage analysis (r^2^ and D’) of the three SNPs in the T2D and control groups. (**B**) Haplotype frequencies of the three loci in both T2D and controls.

Promoter variations within functional genes may contribute to the increase/decrease in promoter activity and biological effects by altering the binding site of the transcriptional factor. In the present study, we explored the promoter mutations of the *SIRT2* gene by DNA pool sequencing, and identified three novel SNPs, including p.-803C/G, p.-770G/C, and p.-166C/A, which were named as P-MU1, P-MU2, and P-MU3, respectively (Figure 1A). The DNA pool sequencing maps and aligning results with GenBank information (NC_000019.10) were displayed in Figure 2B. According to the characteristic of the mutation locus and adjacent sequences, the three SNPs were genotyped by *Msp* I-RFLP, *Alu* I-RFLP, and *Hin*f I-RFLP in all T2D and healthy control individuals, respectively (Figure 1C). The detailed description of the introduced mutation sites and genotyping primers are shown in Table S2.

### 3.2. Linkage Disequilibrium and Haplotype Combination Analysis

The accuracy of imputation to the mutagenic effect is affected by the linkage disequilibrium (LD) of nearby genetic variations or linkage among other indirect SNPs, which may provide further insight into the relationship of several associated SNPs (haplotypes). Therefore, in our study, the LD among the three SNPs of the *SIRT2* gene was examined and evaluated by estimating the r^2^ and D’ values in T2D and healthy control populations. As illustrated in Figure 2A, a strong LD was observed in the T2D group; in detail, P-MU1 and P-MU2 were highly linked according to the r^2^ (0.80) and D’ (0.95), as well as the other two sets of pairwise loci ((P-MU1 vs. P-MU3, r^2^ = 0.82, D’ = 0.94); (P-MU2 vs. P-MU3, r^2^ = 0.72, D’ = 0.94)), exhibiting strong linkage. However, the pairwise sets of the P-MU1, P-MU2, and P-MU3 loci were not in linkage disequilibrium in the control group. In addition, corresponding haplotype blocks were formed due to the highly linked SNPs. All possible haplotypes were constructed, and the distribution frequencies were analyzed in the case and control groups. As shown in Figure 2B, a total of eight haplotypes were observed in all individuals, and the frequencies of H1-GGC and H2-CCA were 57.4% and 34.4%, respectively, which accounted for the most types (91.8%) in T2D patients, while the haplotype frequencies of the two were 14.1% and 26.0% in the controls, respectively. The most dominant haplotype was H5-GGA (27.1%) in the control group; however, it presented 0.8% in T2D population. The results revealed that the LD among the three SNPs and the haplotype distribution presented remarkably different between T2D and non-diabetic subjects.

### 3.3. Population Genetic Analysis and T2D Risk Evaluation

Population genetic information plays an important role in the identification of genetic architectures and multitude effects, especially for exploring the causative locus between the case and control groups. A summary of the genotypic and allelic frequencies, He, and PIC for the three SNPs are shown in Table 1, along with the *P*-value of testing their distribution against Hardy–Weinberg equilibrium. The predominant genotypes were GG (49%), GG (46%), and AA (41%) for the P-MU1, P-MU2, and P-MU3 loci in the T2D population, while their percentages were moderate with 28%, 24%, and 32% in healthy controls, respectively. Further analysis demonstrated that the genotype distributions of the P-MU1 and P-MU2 loci showed a significant difference between T2D and the controls (*p* < 0.01) (Table 2). Further analysis also revealed that the three SNPs presented a moderate He and PIC in T2D or non-diabetic populations. The P-MU1, P-MU2, and P-MU3 in T2D, as well as P-MU1 in the control, tended to significantly differ from Hardy–Weinberg equilibrium (*p* < 0.01), while both P-MU2 and P-MU3 in the healthy control group were in Hardy–Weinberg equilibrium (*p* > 0.05). The significantly different distribution of the haplotype combinations was revealed between two groups (*p* = 0.001, Table 2). To investigate the relationship of the *SIRT2* haplotypes (three SNPs combinations) with diabetes susceptibility, we established the risk estimation analysis and found that the individuals carrying haplotype combinations H1-H1-GGGGCC (OR = 2.03, 95% CI = 1.12–3.72, *p* = 0.02) displayed a significantly increased risk for T2D compared with H2-H2-CCCCAA (OR = 0.89, 95% CI = 0.51–3.24, *p* = 0.16) and H1-H4-GCGCCC (OR = 1.37, 95% CI = 0.69–3.91, *p* = 0.11) (Table 2). The other haplotype combinations in which the number of carriers was lower than five were excluded from the risk analysis and the following studies.

### 3.4. Effects of the SIRT2 Haplotype Combinations on Diabetic Characteristics

**Figure 3:**
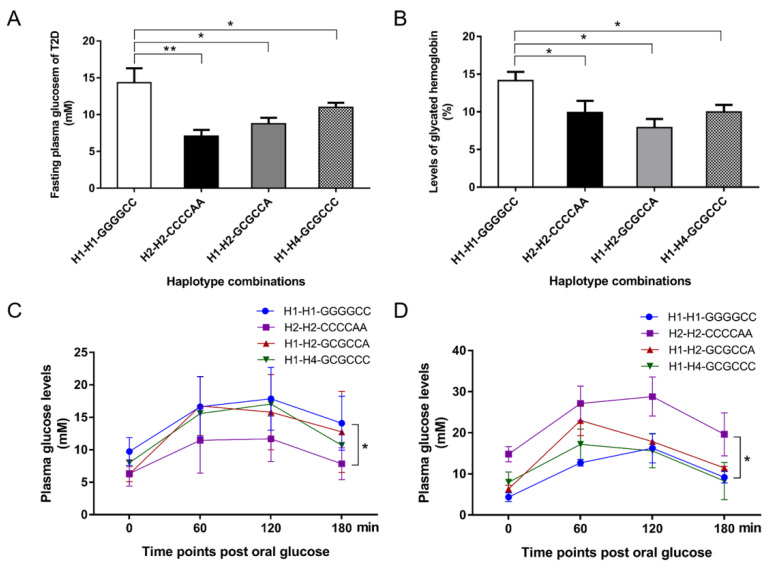
Associations of the *SIRT2* haplotype combinations with clinical characteristics in T2D patients. Effect of haplotype combinations on fasting plasma glucose (**A**) and glycated hemoglobin levels (**B**). Effect of haplotype combinations on glucose content (**C**) and insulin level (**D**) during an OGTT test. Data are given as the mean ± SE. * *p* < 0.05 or ** *p* < 0.01 shows a significant difference.

To examine the role of the linked SNP loci in diabetic pathogenesis, we assessed the associations of the *SIRT2* haplotype combinations with clinical parameters using generalized linear regression analysis. Two indicators, fasting plasma glucose and HbA_1c_, are generally used for the T2D diagnosis. As shown in Figure 3A,B, the individuals carrying H1-H1-GGGGCC had significantly higher values of fasting plasma glucose and HbA_1c_ as compared to those with H2-H2-CCCCAA, H1-H2-GCGCCA, or H1-H4-GCGCCC (*p* < 0.05 or *p* < 0.01). Next, the correlation analysis was established between the haplotype combinations and insulin/glucose level of an OGTT test (Figure 3C,D). The plasma glucose level of the T2D patients with H1-H1-GGGGCC was significantly higher than the ones carrying H2-H2-CCCCAA (*p* < 0.05), as well as showed a high trend comparing to H1-H2-GCGCCA or H1-H4-GCGCCC, while the most reduced insulin of the H1-H1-GGGGCC carriers were found due to the response towards the elevated plasma glucose contents at the 60 min or 120 min timepoint of the test. In addition, the *SIRT2* haplotype combinations were not associated with total cholesterol, triglycerides, LDL, and HDL cholesterol in T2D or the controls (*p* > 0.05, Tables S3 and S4).

### 3.5. Genetic Variation Affects the Promoter Activity by Altering Putative Binding Site of STAT1

**Figure 4:**
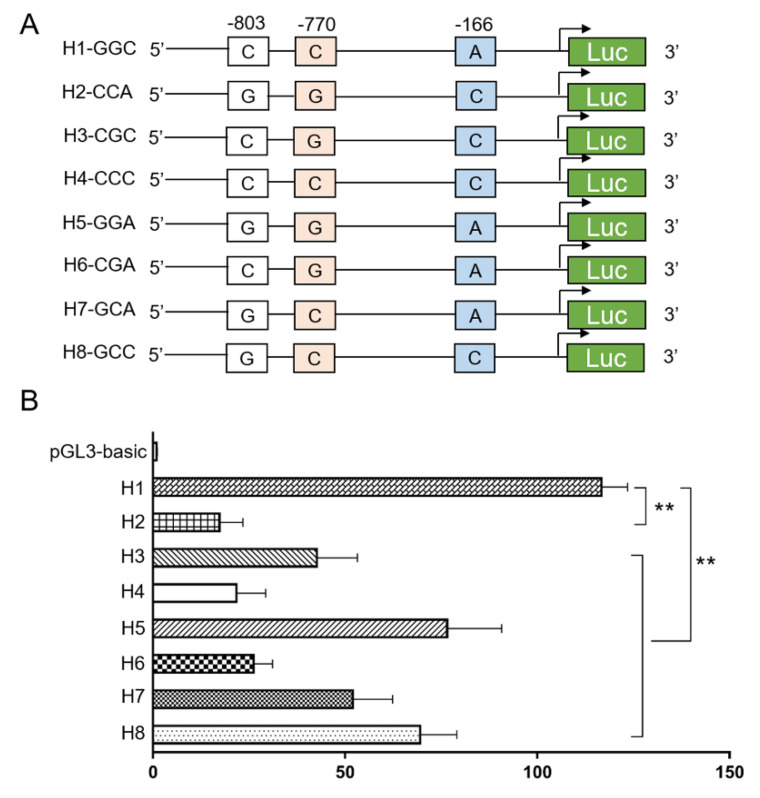
Haplotypes of the SNPs affect promoter activity of the *SIRT2* gene. (**A**) Plasmids with their respective eight haplotypes were constructed. (**B**) Promoter activity of the eight haplotypes were detected by a luciferase assay. Data were given as the mean ± SE. ** *p* < 0.01 shows a significant difference.

**Figure 5:**
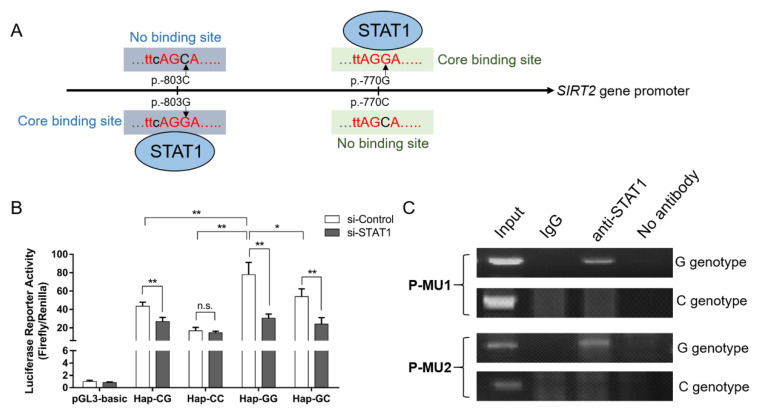
STAT1 can affect the promoter activity by binding to the SIRT2 promoter variants. (**A**) Putative binding sites of STAT1 were disrupted by the P-MU1 and P-MU2 loci. (**B**) STAT1 affected the SIRT2 promoter activity by a reporter assay. (**C**) STAT1 binds to promoter sequences with allele G of the P-MU1 and P-MU2 loci by a ChIP assay. Data were given as the mean ± SE. * *p* < 0.05 or ** *p* < 0.01 shows a significant difference; n.s. represents no significance.

Given the three linked SNPs located in the promoter region of the *SIRT2* gene, we performed a dual-luciferase reporter assay to determine whether the *SIRT2* promoter activity can be affected by the haplotypes. Firstly, we constructed a series of plasmids, which were inserted into the fragments with eight different haplotypes, respectively (Figure 4A). As shown in Figure 4B, consistent with the above association analysis, the plasmid with haplotype H1-GGC presented the highest promoter activity than the others, and differed up to 6.74-fold as compared to haplotype H2-CCA, which suggested that the transcriptional activity of the *SIRT2* gene was positively associated with diabetic susceptibility. To further explore how the promoter activity was affected by the genetic variations, we predicted the possible transcriptional factors binding to the mutation sequences, and found that the binding sites of signal transducer and activator of transcription 1 (STAT1) were disrupted at the P-MU1 and P-MU2 loci (Figure 5A). Next, the effects of STAT1 on the promoter activity with four different haplotypes (combined by P-MU1 and P-MU2) were detected by a reporter assay. As shown in Figure 5B, the haplotype Hap-GG displayed the highest promoter activity than the other three haplotypes. Knockdown of STAT1 can significantly reduce activity in the Hap-GG, Hap-GC, and Hap-CG groups. However, the promoter activity of the plasmid with Hap-CC was not affected by the STAT1 silencing. In addition, the ChIP assay was performed and validated that STAT1 could directly bind to the promoter sequences with allele G of both the P-MU1 and P-MU2 loci (Figure 5C).

## 4. Discussion

Genetic predisposition is considered as the most prominent factor for T2D occurrence and development [20]. Thus, exploring the functional genetic variation, especially the specific locus associated with T2D, can provide potential markers for clinical diagnosis and drug target design. Dysregulation of glucose metabolism, insulin sensitivity and beta-cell function are the hallmarks of T2D physiology and pathogenesis [21]. As a highly conserved NAD^+^-dependent histone deacetylase, SIRT2 participates in many metabolic processes, such as oxidative stress, mitochondrial efficacy, and insulin resistance [9,22]. In our study, we discovered three novel strongly linked SNP loci in the *SIRT2* promoter region, and their corresponding haplotypes affected the transcriptional activity of the *SIRT2* gene, finally resulting in the abnormal serum indexes (fasting plasma glucose, glycated hemoglobin and insulin secretion) of T2D patients. Our findings expanded the possible application scope of the *SIRT2* variants in human disease.

Functional SNPs in strong linkage disequilibrium of the same chromosome are organized into a cluster of haplotype blocks that reflect the possible hotspots of recombination [23,24]. Haplotype structure construction has been widely performed in genomic selection and genome-wide association studies, and is considered as a coherent way to explore the significance of SNP integration compared to a single SNP assay [25,26]. Shen et al. established the multi-allelic haplotype association and identified a new protective haplotype of the *LRP8* gene for coronary artery disease and myocardial infarction, which was not revealed in their previous single-SNP analysis [27]. We detected three highly linked SNPs in the *SIRT2* promoter region, and the combined haplotypes were significantly associated with clinical indicators of T2D patients. In recent years, extensive studies were demonstrated to screen genetic variations in the promoter regions of functional genes due to the more mutable sites of the regulatory region than the conserved coding sequence. Promoter mutations may contribute to the alteration of transcriptional activity, expression dose, and phenotypic characteristics [28,29]. Kwon et al. found that the *OCT3* promoter haplotype H2 significantly affected luciferase activity as well as the metformin pharmacokinetics [30]. Our previous study reported that the T-A haplotype of the *CREB1* gene exhibited maximal promoter activity, and resulted in susceptibility to T2D [14]. Consistently, in this study, the haplotype H1-GGC of the *SIRT2* gene is associated with increased transcriptional activity and high diabetic risk. Studies, such as the effects on gene function, the mechanisms of haplotype regulating the signaling pathway, and pathogenesis, need further investigation.

From the luciferase assay and association analysis, we conclude that the increased SIRT2 level is positively correlated with T2D risk and development, which is attributed to the roles of the *SIRT2* gene in biogenesis and functional regulation of glucose and insulin. Inhibition of SIRT2 significantly decreased glucose output in a dose-dependent manner by hyperacetylation of PEPCK1, a critical enzyme for gluconeogenesis [31]. Amita et al. determined the role of SIRT2 in insulin-mediated glucose disposal in skeletal muscle cells, and revealed that downregulation of SIRT2 can improve insulin sensitivity under insulin-resistant conditions [32]. Correspondingly, the etiological effects of the *SIRT2* haplotypes on glucose content and insulin action were verified in our study. In addition, several genetic polymorphisms of the *SIRT2* gene have been identified in Chinese populations. For instance, an SNP locus (rs2241703) in the 3’UTR of the *SIRT2* gene, which disrupted the binding site of miR-486-3p, was associated with the risk of Parkinson’s disease [33]. Another polymorphism rs2241703 of *SIRT2* was reported to affect Alzheimer’s disease [34]. For T2D, Liu et al. found three respective SNPs increased the transcriptional activity of the *SIRT2* gene [35]. Comparing to this single-SNP study, we demonstrated the composite effect of haplotypes from three highly linked SNPs on *SIRT2* transcription and T2D risk, which suggested a promising combined application of the causative variants in T2D diagnosis and therapy.

Promoter mutations have their most probable effects on the transcriptional activity of the corresponding gene by altering the binding sites of regulatory enhancers or suppressors, which consequently affect the gene expression and then result in the occurrence and development of human disease [36]. After bioinformatics analysis and experimental verification, we found that the C allele of the P-MU1 an P-MU2 loci caused disruption of the binding site of STAT1, and a decreased SIRT2 level and impaired T2D risk were observed compared to the G allele. Recently, an atlas of cis-regulatory modules with allelic variation was generated to determine the transcription factor (TF) binding affinity in human lymphoblastoid cell lines, and >1300 TF-binding variants were revealed in association testing [37]. Li et al. exploited the CRISPR interference to correct the -124C>T promoter SNP of *TERT* to -124C allele. This modification significantly reduced TERT transcription and induced the senescence of gliomas cells [38], which presented the opposite results to our study for deciphering transcriptional control. STAT1 was reported to play regulatory roles in the pathogenesis of diabetic complications, including diabetes with cardiovascular disease and diabetic nephropathy [39,40]. Upregulated expression of STAT1 was associated with endothelial dysfunction, and was validated as a molecular marker for maternal and fetal T2D [41]. Moreover, a previous study reported that SIRT2 functions as an inducible factor in angiogenesis by regulating the secretion of VEGFA [42]. All these suggest that STAT1 may participate in the metabolic processes of glucose and insulin by enhancing SIRT2 for T2D or microvascular complications. Interestingly, considering the promoter activity differences between the pairwise sets of H2 vs. H4 and H1 vs. H5, we found that the C allele of P-MU3 accelerated the *SIRT2* transcription comparing to the A allele. However, in our study, no putative transcription factor was predicted in the P-MU3 locus by bioinformatics analysis. In further studies, the protein mass spectrometry assay and ChIP-seq technology may be combined to screen the contributing enhancer or attenuator involved in the P-MU3 mutation.

In conclusion, this is the first study ascribing the effects of haplotype combinations (three strongly linked SNPs loci) within the *SIRT2* promoter region on transcriptional activity and diabetic characteristics in the Chinese population. Divergent haplotype distributions were detected between the T2D and healthy controls (Table S3 and Table S4). The haplotype H1-GGC significantly increased the *SIRT2* promoter activity, and the individuals carrying its homozygous combination H1-H1-GGGGCC displayed a higher fasting plasma glucose and glycated hemoglobin, as well as a lower insulin level than the other groups. We also proved that the variants could affect *SIRT2* transcription by disrupting the binding sites of STAT1. Further in vitro and in vivo investigations are required to fully verify the role of the *SIRT2* promoter polymorphisms using a larger-scale population. Nevertheless, our study suggested that the haplotype H1-GGC was the induced factor for T2D susceptibility, and in the future could be expected as a molecular marker for clinical diagnosis, therapy, and clustered genetic assessments of T2D.

## Figures and Tables

**Table 1:** Population genetic information of the *SIRT2* SNPs in T2D patients and healthy controls.

Loci	Group	Genotypic Frequencies Group: T2D (209), Control (223)				Allelic Frequencies		He	PIC	HWE (*p*) ^1^
P-MU1 (p.-803C/G)		CC	CG	GG	*p*-value	C	G			
	T2D	44 (21%)	63 (30%)	102 (49%)	0.002 **	36%	64%	0.46	0.35	<0.01
	Control	76 (34%)	85 (38%)	62 (28%)		53%	47%	0.50	0.37	<0.01
P-MU2 (p.-770G/C)		GG	GC	CC	*p*-value	G	C			
	T2D	95 (46%)	65 (31%)	49 (23%)	0.001 **	61%	39%	0.48	0.36	<0.01
	Control	54 (24%)	100 (45%)	69 (31%)		47%	53%	0.50	0.37	>0.05 a
P-MU3 (p.-166C/A)		CC	CA	AA	*p*-value	C	A			
	T2D	48 (23%)	75 (36%)	86 (41%)	0.194	41%	59%	0.48	0.37	<0.01
	Control	49 (22%)	103 (46%)	71 (32%)		45%	55%	0.50	0.37	>0.05 a

^1^*p* (HWE)-value with “a” representing a group in Hardy–Weinberg equilibrium. He, heterozygosity; PIC, polymorphism information content. ** *p* < 0.01 shows a significant difference.

**Table 2:** Distribution differences of the *SIRT2* haplotype combinations and the risk of type 2 diabetes.

Loci	Haplotype Combinations	T2D (*n*)	Control (*n*)	*p*-Value ^2^	Odds Ratio	95% CI
p.-803C/G p.-770G/C p.-166C/A	H2-H2-CCCCAA	40	12		1	
	H1-H1-GGGGCC	85	29	0.02 *	2.03	1.12–3.72
	H1-H2-GCGCCA	57	42	0.16	0.89	0.51–3.24
	H1-H4-GCGCCC	7	14	0.11	1.37	0.69–3.91
	*p*-value ^1^	0.001 **				

^1^ The *p*-value shows the different distributions of the haplotype combinations between the T2D and control groups, and the value was assessed by Yates’ correction of the Chi-square test. ^2^ The *p*-value shows the risk to T2D of the haplotype combinations, and the value was assessed by a Chi-square test or Fisher’s exact test. * *p* < 0.05 or ** *p* < 0.01 shows a significant difference.

## Supplementary Materials

The following are available online at https://www.mdpi.com/2073-4425/11/5/569/s1, Table S1: Clinical characteristics of subjects in this study. Table S2: Primers used in this study. Table S3: Associations of the haplotype combinations with clinical traits in T2D. Table S4: Associations of the haplotype combinations with clinical traits in healthy controls.
