# Supplementary material for: Haplotypes of the Mutated SIRT2 Promoter Contributing to Transcription Factor Binding and Type 2 Diabetes Susceptibility
Source: Genes (Basel). 2020 May 19;11(5):569. doi: 10.3390/genes11050569 (PMC7288287; doi:10.3390/genes11050569)
Supplement: Supplementary file 1 [file genes-11-00569-s001.pdf]

Table 1. Clinical characteristics of subjects in this study.

Clinical characteristics	T2D	Healthy controls	*P* value
Number (male/female)	209 (103/106)	223 (131/92)	-
Age (years)	54.15±10.32	50.24±8.53	Not significant
Weight (kg)	66.26±11.09	62.50±10.47	<0.05
BMI (kg/m^2)	23.78±2.17	21.72±3.61	<0.01
Fasting glucose (mM)	6.23±2.42	5.06±0.14	<0.01
HbA$_{1c}$ (mmol/mol (%))	7.90±3.53	-	Not determined
Total cholesterol (mM)	5.26±1.11	4.78±0.56	<0.05
Triglycerides (mM)	1.89±0.65	1.24±0.29	<0.01
Low density lipoprotein (mM)	2.91±1.03	2.59±0.63	<0.05
High density lipoprotein (mM)	1.13±0.48	1.39±0.73	<0.01

The *P* values compared clinical and laboratory data between T2D and controls by using Student's unpaired *t*-test. $P<0.05$ and $P<0.01$ showed significance and high significance, respectively.

Table S2 Primers used in this study.

Primer name	Gene ID (22933)	Primer sequences	Tm (^{o}C)	Production sizes (bp)
Primers for detecting mutation	SIRT2-promoter	F1: GTCAGAACAGAGACCAATTG<br>R1: GTGACGGCACCAGAAATGGG	56.3	1675
Primers for *Msp* I-RFLP	p.-803C/G	[a]F2: GGTTAGAGGTCAGGAAGTTCCG<br>[a]R2: TGCCAAACGGAGCCCTAGGACT	58.5	118
Primers for *Alu* I-RFLP	p.-770G/C	[a]F3: ATGATTGGGTTAGGATCATAGAT<br>[a]R3: TTAATCTCTGATCCCTGAGGCTA	55.7	140
Primers for *Hinf* I-RFLP	p.-166C/A	[a]F4: AAAGCGCGTCTGCGGCCGCAGAG<br>R4: TCTGTCCCGTCACCAACCAC	55.1	151
Primers for constructing pGL3	SIRT2-promoter (containing the 3 SNPs)	[b]F5: CTGCTAGCGGTTAGGATCATAGCTAG<br>[c]R5: CCAAGCTTCCGACTGCTCTGTCCTGT	58.1	824

a, the underlined base showed mismatches to create restriction sites.

b and c, the underlined base showed the restriction sites of *Nhe* I and *Hin*d III, respectively.

Table S3 Associations of the haplotype combinations with clinical traits in T2D.

Loci	Haplotype combinations	Clinical traits (Mean ± SE)			
		TC (mM)	TG (mM)	LDL (mM)	HDL (mM)
**p.-803C/G p.-770G/C p.-166C/A**	H1-H1-GGGGCC (85)	6.46±0.66	1.47±0.29	2.91±0.24	1.29±0.35
	H2-H2-CCCCAA (40)	3.94±0.37	1.69±0.32	2.72±0.17	1.41±0.15
	H1-H2-GCGCCA (57)	5.55±0.18	1.85±0.35	3.23±0.26	1.62±0.69
	H1-H4-GCGCCC (7)	4.85±0.62	2.04±0.79	2.74±0.51	1.22±0.17
	P-value	0.46	0.71	0.56	0.80

TC: Total cholesterol; TG: Triglycerides; LDL: Low density lipoprotein; HDL: High density lipoprotein.

Table S4 Associations of the haplotype combinations with clinical traits in healthy controls.

Loci	Haplotype combinations	Clinical traits (Mean ± SE)			
		TC (mM)	TG (mM)	LDL (mM)	HDL (mM)
**p.-803C/G**	H1-H1-GGGGCC (85)	4.32±0.81	1.23±0.16	2.26±0.41	1.07±0.22

**p.-770G/C p.-166C/A**	H2-H2-CCCCAA (40)	4.64±0.42	1.41±0.25	2.73±0.32	1.34±0.27
	H1-H2-GCGCCA (57)	3.57±0.37	1.03±0.13	2.38±0.35	1.40±0.31
	H1-H4-GCGCCC (7)	3.91±0.75	0.98±0.12	2.81±0.49	1.08±0.19
	P-value	0.17	0.34	0.76	0.18

TC: Total cholesterol; TG: Triglycerides; LDL: Low density lipoprotein; HDL: High density lipoprotein.
